# Towards a framework for multisector and multilevel collaboration: case of HIV and AIDS governance in South Africa

**DOI:** 10.1080/16549716.2019.1617393

**Published:** 2019-06-03

**Authors:** Pinky Mahlangu, Jane Goudge, Jo Vearey

**Affiliations:** a Gender and Health Research Unit, South African Medical Research Council, Cape Town, South Africa; b School of Public Health, Faculty of Health Sciences, University of Witwatersrand, Johannesburg, South Africa; c African Centre for Migration & Society, School of Social Sciences, University of Witwatersrand, Johannesburg, South Africa

**Keywords:** Multisectoral action, HIV response, framework, collaboration, South Africa

## Abstract

**Background**: While multisectoral action (MSA) is advocated as one of the strategies to address complex health and development challenges, there is limited clarity about the process of multisector collaboration in practice.

**Objectives**: Informed by the findings of the research on implementation of the multisectoral response to HIV in South Africa, and drawing from the existing literature; we propose a framework for multisector and multilevel collaboration. The framework describes key components of the process of multisector collaboration, and aims to inform policy and practice.

**Methods**: An integrative review and synthesis of existing frameworks, models and approaches on multisectoral action in public health, governance and health, and in public administration was conducted to inform the development of the proposed framework.

**Results**: There are seven key components that are critical in the process of multisector collaboration namely: preconditions; key drivers; structure; mechanisms; administration; execution and evaluation. Multisector collaboration is presented as an iterative process that allow for improvement and learning. The framework is presented through a visual representation which shows how the seven elements are connected, and how learning happens through-out the multisector collaboration process. Structure and mechanisms are the two central and interrelated elements of the proposed framework.

**Conclusion**: The framework does not suggest that multisector collaboration is a panacea, but that MSA remains critical to address complex health and development issues. Focus should be on finding innovative ways to inform and strengthen its implementation in practice. The framework can be used by practitioners and policy makers to inform design, implementation, and evaluation of multisector collaborations. It reflects on complexities of MSA, and brings to the fore critical information to assess readiness and to inform the decision whether to engage in MSA or not.

## Background

A multisectoral approach (MSA) refers to an approach or a tactic to address a problem from multiple angles that involve various sectors of society involved in governance, namely government, civil society, the private sector, community structures, and individuals [1,2]. Such an approach is required when a problem which is being addressed is beyond the scope and resources of a single sector [3,4]. MSA addresses important contextual factors like the social determinants of health that, if ignored, can increase health inequalities [1]. To enact such an approach, multisector collaborations (MSCs) – also referred to as cross-sector collaborations – are established [5,6]. Multisectoral collaboration refers to a partnership involving two or more sectors (e.g. government, civil society, private sector, community group or individuals) who come together to solve a complex societal problem that results from multiple determining factors and cannot be addressed by one sector acting alone [7]. Multisector collaboration and multisector action refers to a similar process, and are used synonymously.

Multisector collaborations have increasingly become a necessary and desirable strategy for addressing health and development challenges, including HIV and AIDS [6,8,9]. They provide a platform for including major stakeholders in society in the development of interventions, supporting information sharing and coordination between sectors, and – crucially – the sharing of responsibility [10–12]. Multisector collaboration also allows for innovation, greater responsiveness to complex situations; and leveraging knowledge, expertise, reach, and resources from combined and varied strengths of multiple sectors [1,10,13]. Notwithstanding it’s benefits, multisectoral collaboration is complicated and challenging to implement [14–16]. Multisectoral collaboration brings together people with different background, strategies and tactics; and members come with or represent competing institutional logics (rules and standard operating procedures) based on their institutional background [5,17,18]. These collaborations can be costly in time and resources, and are inherently fragile, requiring constant management, coordination, proper planning, reciprocal obligations and trust between sectors [19,20]. There is a likelihood of conflict, often associated with shifting of responsibility, and the power dynamics inherent in multisector collaborations [21,22].

Multisectoral collaborations are not new, theoretical and empirical research on this subject has proliferated, particularly in public administration, health and development literature. Wood and Gray [23] are amongst the pioneers who have influenced the theory and practice of collaboration. Since then, frameworks and models on multisector collaborations have been developed [5,11,15,22,24–27]. Deliberation on approaches to address health through multisectoral action has also flourished within public health research and practice [1,8,9]. Furthermore, multisectoral governance for health has become a key area of interest [3,10,28,29]. Most recently, Bryson, Crosby [6] synthesised the literature on collaborations and found that though much has happened, much has not changed. Multisector collaboration remains a complex phenomenon. Whilst the case for multisectoral collaboration has been widely established, there has been limited clarity about the process of collaboration [6,18]. Focus has been mainly on describing key requirements that determine success and on the outcome of multisector collaboration; with limited explanation of the process and its execution in practice – particularly – when multiple sectors are involved at multiple levels of government. The challenge remains to better understand and find ways to effectively implement multisectoral action that will produce good results and minimise failure [6,9].

The objective of this study was to explore the extent to which existing frameworks inform and explain MSA implementation in practice. The review was conducted to highlight where there is not sufficient evidence in the area of MSA implementation, to refine the literature and fill the knowledge gaps. Building on the research documented in two previously published papers, and informed by the review of extant literature, we propose a framework for multisector and multilevel collaboration. We first provide background about the envisaged process of multisectoral collaboration in the case of HIV in South Africa, and summarise the challenges experienced in practice. We describe the methods used to conduct the review of existing frameworks, models and approaches on multisectoral action, and discuss the findings from conduct the review. We then draw on the South African experience to contribute to the existing literature, through development of a framework for multisectoral action; and lastly discuss the implications of the framework to theory, policy practice.

## Multisector collaboration on the HIV response in South Africa

In South Africa, MSA – as a central tenant of the national response to HIV – has evolved over time, reflected in national HIV plans since 1994. The National AIDS Plan of 1994 emphasised inclusion of non-state actors, as well as establishment of multisectoral structures to coordinate implementation of the country’s response to HIV at national and provincial level [30]. The HIV/AIDS and STI NSP for South Africa 2000–2005 reiterated the key principles of cooperation and inclusion and establishment of a national multisectoral coordinating structure, South African National AIDS Council (SANAC) to drive programme implementation [31]. The concept ‘MSA’ was formally referred to and incorporated in the NSP (2007–2011) which acknowledged that government alone will not be able to develop and implement a comprehensive response to HIV and AIDS, highlighting the need to include non-government actors in the HIV policy process [32]. The MSA continues to be amongst the key principles framing the NSP 2012–2016, and the NSP 2017–2022 [33,34]. It is envisaged that through multisector collaboration, sectors including government, civil society and the private sector in South Africa will work together, including pooling resources, knowledge and expertise to address the complex social and structural drivers of the epidemic of HIV and AIDS [32]. Collaboration of sectors happens through AIDS Council structures that have been established at national, provincial and local level, mandated to work towards a common goal – a South Africa free from the burden of HIV – stipulated in the National Strategic Plan (NSP) on HIV and AIDS. While AIDS Councils are a government mandated vehicle for multisector collaboration, there is no framework informing how they should operate. A whole of government approach to HIV is also envisaged as part of multisector approach to HIV; with all government departments expected to mainstream HIV in their policies and plans.

The review of implementation of the multisectoral response to HIV was undertaken through a research conducted with AIDS Councils and government departments in South Africa. The study highlighted that these structures experience challenges in implementing a multisector approach. Detailed methods and full presentation of findings are discussed elsewhere [35,36]. Tables 1 and 2 presents a summary of the challenges.

**Table 1.** Challenges impacting on effective implementation of multisector collaboration.

	Challenges amongst AIDS Councils	Lessons
Composition	There is no guidance regarding establishment of AIDS Councils, except that they need to be multisectoral Some of the critical sectors are not represented, while some struggle to define their role Membership is continuously changing AIDS Councils struggle to mobilise stakeholders that need to be involved	Structure is important for effective functioning of the collaborative Buy-in from all sectors is critical for participation and commitment Membership should be informed by a good stakeholder analysis to determine which sectors should be involved, resource needed and the capacity they have that will benefit the collaborative
Operation procedures	The structure relies on a person, the HIV Coordinator, and ceases to function when he or she resigns Lack of decision-making power amongst members, limiting ability to commit or implement resolutions Roles and responsibilities between sectors and amongst members are not defined There is limited capacity amongst members to strategically plan, monitor and report on progress Meetings happen on an ad hoc basis There is no pre-defined plan to inform day to day operation of the structures (e.g., communication, resolving tension)	Administration of the collaborative should be institutionalised and not left to an individual, for continuity Having a leader, someone with influence, legitimacy and respect is critical for credibility of the collaborative A predefined plan with clear activities, and standard operating procedures should inform the process of collaboration
Relations between sectors	There is tension between sectors, with some being undermined because of differences in institutional logic (norms, and practices) There are power issues linked to contribution of resources (the one contributing financial resources viewed as more powerful compared to other sectors) Prior history of adverserialism There are divisions and lack of trust between sectors	Trust, equality and respect for others are some of the key process elements critical to accomplish the goals of the collaborative A communication and conflict resolution strategy must be put in place should such issues arise
Institutional challenges including poor leadership and support	The national level structure is caught up in strengthening itself as an organisation, and neglected its oversight role of capacitating & supporting sub-level structures Coordination of the multisector response is an unfunded mandate, members take own money to pay for activities of the AIDS Council There are many institutions involved at national level, with overlapping roles and responsibilities which are not harmonised	National guidance and availability of resources (financial and human) are critical pre-requisites, which determines the outcome of the collaborative
Evaluation	There is lack of defined systems to measure the impact of the collaboration There is poor tracking of the contribution of the private sector	Monitoring and evaluation and continuous learning are an important part of the process Oversight, and clarification of responsibilities is critical for accountability

**Table 2.** Challenges of mainstreaming HIV in health and non-health departments.

	Challenges in government departments	Lessons
Mainstreaming	Most departments have workplace policies, but some non-health departments are unclear on how HIV aligns with their core (service delivery) mandate. The NSP does not provide a framework to inform the mainstreaming process	Clarity on individual role and contribution to the collective is a critical structure element that has an influence on performance and outcome
Coordination between levels	HIV programmes within some department were planned and implemented in isolation Lack of a planned processes or systems to identify synergies and ensure that directorates work together Some directorates were prioritising their activities over those of others	Integrated planning and implementation of progammes is a key component of the collaboration process
Coordination across departments	There is a wide recognition of the need for a ‘whole of government’ response to HIV amongst departments; yet limited evidence of collaborations on HIV and AIDS programmes between departments Roles and responsibilities are overlapping	Coordination of efforts and working towards a collective goal versus sector goals is needed for effective use of resources and for collaboration
Systems for intergovernmental relations	Some departments are not represented, and some not regularly participating in multisector platforms meant for coordinated planning and collaboration	Multisector collaboration also requires regular interaction to identify synergies and areas of collaboration
Evaluation	Lack of clarity regarding how the outcome will be measured	Monitoring and evaluation should be defined from the beginning when objectives are set, with continuous monitoring through-out the process, and evaluation at the end of the process
Reporting	Reporting system at national level involves multiple actors (South African National AIDS Council, Department of Planning Monitoring and Evaluation and Department of Public Service and Administration) and is poorly coordinated	Harmonised and integrated system of reporting is critical to ensure that there are no omissions and duplication in reporting.

## Methods

An integrative review was undertaken to explore how existing frameworks on MSA explain and inform MSA implementation in practice. The review was undertaken to provide evidence in area of MSAP implementation process [37,38].

### Search strategy

The literature on multisector collaboration was reviewed with particular interest on existing frameworks and models on multisector collaboration. Other terms that are synonymously used to refer to multisector collaboration were noted in the literature and included cross sector collaboration, collective planning, collaborative governance, and collective impact. The literature search was conducted in Pub Med, Google scholar and Scopus. The search terms used was multisector collaboration framework AND/OR model, multisectoral collaboration framework AND or model, cross sector collaboration framework AND/OR model, collaborative governance framework AND/OR model, collective impact framework AND/OR model, multisector governance framework AND/OR model, and multisectoral action framework AND/OR model. We also reviewed reference lists of key papers that we found to search for articles on multisector collaboration frameworks and models.

### Inclusion and exclusion criteria

The search was restricted by year of publication from 2006 to 2018, and articles with full text and in English were reviewed. Other than the work of Wood and Gray [23] and that of others, 2006 onwards is the time when frameworks on collaborative action started to emerge in numbers. Only the MSA frameworks published in peer reviewed journals were included in the review. We excluded unpublished frameworks in dissertations, grey literature and in conference abstracts. We included MSA frameworks that reflected on implementation of the multisectoral approach in practice, while we excluded frameworks aimed to inform development of multisectoral action plans, and on MSA evidence.

Two independent reviewers (PM and JG) were involved to assess the quality of the reviewed framework. All articles were screened on title, abstract and key words by two reviewers and were included if the title, abstract, key words indicates that it is a framework, model or approach on multisectoral action, multisector collaboration, collaborative action, and collaborative governance. After the initial selection, the remaining articles were screened full text by two reviewers, and included if the article focuses on implementation of MSA in practice. Where there was a disagreement about whether to include or not include a framework, a third person (JV) was involved to assist in the decision-making process. JVs involvement was only required once, where it was not clear whether the framework was on MSA implementation or not.

**Figure 1.**
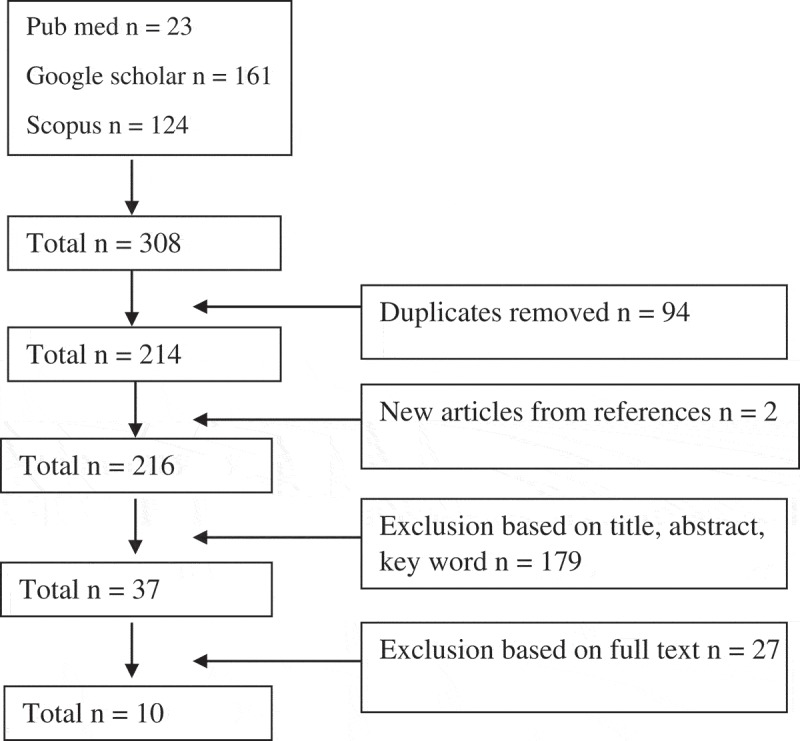
Flow chart of the integrative review.

In total 308 articles were identified, 94 were removed because they were duplicates (See Figure 1). Using a search of reference lists of included papers, two more papers meeting the inclusion criteria were identified. A total of 179 papers were excluded after reviewing the title, abstract and key word where we found that the framework describes how to develop an MSA plan, was a dissertation, or did not have full text, or not in English. After reviewing the full text papers, nine frameworks that met the inclusion criteria – focusing on implementation of MSA – were included in the review.

### Data analysis

Gray and Wood [39] defines three broad issues that are essential to understanding collaboratives [1]: the preconditions that make collaboration possible and motivate stakeholders to participate [2], the process through which collaboration occurs, and [3] the outcome of the collaboration. The review of existing frameworks on multisector collaboration focused on the three components defined by Gray and Wood, and further expanded to include a reflection on how the concept of collaboration is defined, focus and aim of the framework, reflection on the structure, description of essential elements in multisector collaboratives, and also highlighted the contribution and the gaps. The term element is used in this paper to refer to a component or a part that makes-up a framework.

## Review of existing frameworks, models and approaches to multisectoral action

Table 3 presents a synthesis of the review of existing frameworks, models and approaches on multisector action [1,3,5,8,9,11,24–26]. All the frameworks reviewed (See Table 3) make a meaningful contribution on multisectoral action, with each placing emphasis in some areas and not in others. Substantive work has been done, focusing on defining the concept, discussing the preconditions and prerequisites (inputs) and the outcomes of multisectoral action [6,40].

**Table 3.** Synthesis of existing frameworks, models and approaches to multisectoral action.

	Model of collaborative governance [11]	Integrative framework for collaborative governance [24]	Collective impact framework [25]	Framework for cross sector collaboration [5]	A process framework of collaboration [26]	Multisector approach for public health [1]	Governing multisector action for health [3]	Multisector governance for health [9]	Multisector action for health [8]	Multi-sectoral approach to NCD Prevention [41]
Definition of MSA	A governing arrangement where public agencies engage non-state actors in a formal collective decision making	Public policy structures engage levels of government, private and civic spheres to carry out a public purpose	Long-term commitment by different sectors to a common agenda to solve a specific social problem	Partnership involving government, business, nonprofits and philanthropies, communities	Autonomous actors interact through formal and informal negotiation	Deliberate collaboration among various stakeholders to jointly achieve a policy outcome	Brings together various actors sectors to achieve a common goal	A governance mechanism where government and non-government actors solve multidimensional health challenges	Actions undertaken by non-health sectors to protect the health of the population	Involvement of any 2 or more sectors, one of which must be government.
Focus and aim	Visual representation of four elements: starting conditions, institutional design, facilitative leadership and collaborative process, together producing outcomes	Visual representation of five elements: system context, drivers, collaborative governance regime, and its internal collaborative dynamics and collaborative actions that generate impacts and adaptation	Tables five conditions of collective impact: common agenda, shared measurement, mutually reinforcing activities, continuous communication and backbone support	Visual representation of initial conditions, process, structural governance components, constraints and contingencies, outcomes and accountability	Describes five dimensions to be known for effective collaboration: governance, administration, organisational autonomy, mutuality and norms	Describes a conceptual framework, including advantages, prerequisites and bottlenecks, process and outcomes with examples of application	Identifies five key considerations and lessons for successful governance of multisectoral action	Illustrates the complexity of multisectoral governance in real practice	Identifies the challenges, opportunities and capacity development needed for effective multisectoral actions for health in a complex policy environment	Tabulated framework for MSA to Health Policy Analysis made up of four main constructs: context, content, stakeholders, and strategies
Preconditions and prerequisites	Prior history of conflict or cooperation, the incentives for stakeholders to participate, power and resource imbalances, facilitative leadership and institutional design	leadership, consequential incentives, interdependence, and uncertainty, and context which impacts on the success (or not)	An influential champion, adequate financial resources, and a sense of urgency for change.	Precondition for collaboration: general environment, sector failure and direct antecedents	Time, respect for the fragility of the process, and paying close attention to the process will increase the likelihood that success will occur	Willingness at the leadership and mandate at the policy level, sufficient resources and time, open discussion, communication, monitoring and assessment	Distributed leadership, issue framing, negotiation skill, flexibility and learning, communication, and relationship building, ethics and integrity, and institutionalised structure	Sharing a common goal or vision, negotiation skills, good governance, government bureaucracy delays implementation	Mutual gain, consensus across all partners to reach a shared vision, external driving forces, trust among, balance between soft power and legal, and good governance	Establishing means for engagement; funding through joint budgeting; complementary vision; establishing interests and concerns of actors and a government led process are some of the key factors in a policy process
Structure		Focus on who the right people/members should be in collaboratives		Specialisation of tasks and division of labour, rules and standard operating procedures, and designated authority relations		clear roles and responsibilities, an institutional mechanism, coordination committee, channels for regular communication, and measures for problem solving	Leadership role defined, clarity of roles of sectors, mapping of the profile, interests, incentives, and relationships of key individuals and sectors, not depend on an individual	Appoint a lead agency to do internal coordination, balance of power and interest amongst stakeholders, joint financing and monitoring	capacities at individual, institutional and system	Clarification of roles of actors (sectors) in government and outside of government is critical in a policy process
Process	cyclical or iterative rather than linear, communication, face to face dialogue, trust building, commitment to the process and shared understanding	Discovery, definition, deliberation, and determination, sees collaboration as an iterative process	Three phases of getting a collective impact: initiate action, organise for impact, and sustain action and impact.	Process includes forging initial agreements, building leadership, building legitimacy, building trust, managing conflict, and planning.	Collaboration occurs over time as organisations interact formally and informally through repetitive negotiation, & execution of commitments	Strategies and procedures to be defined before implementation All stakeholders need to be made part of decision‑making to make it participatory	governing implementation processes requires explicit attention up front. Requires accountability, transparency, trust, innovation, adaptation, and flexibility			The framework refers to strategies (intentional choices maximising benefits) instead of process, means of engagement of other sectors includes consultations, workshops, or meetings
Outcome	Small wins deepen trust and are critical outcome measure in collaboratives	Results on the ground are an important outcome indicator	Progress happens along the way. Early wins that demonstrate the value of working together are essential to hold the collaborative together.	Differentiates between three categories: public value; first-, second-, and third-order effects: ‘public value’ should be the point of creating and sustaining cross-sector collaboratives		MSA plan has to be defined, monitoring and supervision are to be jointly planned for coordination	Imperative to cultivate a culture of mutual learning among the divergent stakeholders, a shared mental model such as theories of change or logic or outcome models is key		Progress should be monitored through regular reports which are publicly available	
Contribution	Conditions under which collaborative governance will be more or less effective, approaches collaborative process as a type of governance. which is easy to understand	Interaction of context (environmental factors) and internal dynamics and actions in collaborations influences outcome. The 10 propositions offered are useful to enquiry and development of theory.	It is realistic about the time factor in collaboratives – describing that collaboratives requires time and should not be undertaken as a quick fix solution	Deliberates on both process and structure, and the challenging nature of collaborating, and makes critical pointers on how to approach failure	The listing of the key process element in collaboratives has been a key contribution of the framework, starting to fill ‘the black box’ the process of collaborating.	Deliberates on the possible positive outcomes/benefits of MSA				A useful framework (for a multisectoral approach) to health policy analysis. It is a useful guide in thinking about the different actors (sectors), their interest and agenda, and their role in the policy process

There has been more focus on the design of multisector collaborations and what they require, with very limited reflection on the implementation process and its challenges. All except three frameworks [5,26,41] offer an explanation of the process of collaboration. Except for the three frameworks [5,26,41] and the approach defined by Salunke and others [1], others simply list the process elements needed in multisector collaborations, for example, building trust, managing conflict, and need to define procedures before implementation. While these frameworks list the process elements, there is limited reflection on what needs to be done to realise the listed process elements, the ‘how’ of the process of multisector collaboration. This limitation according to Wood and Gray [23] is the ‘black box’, and a least understood part of collaboration [26].

As an important element related to the process of collaboration Bryson, Crosby [5] discussed the concept of structure in multisector collaborations. They argue that structure has not attracted much interest in the literature, in part because researchers have emphasised organising as a process over organisation. As shown in the proposed framework and noted by Bryson, Crosby [6], structure and process are both important and interrelated. Design of structure has attracted interest in public health literature, with focus on definition of roles and responsibilities, highlighting the need for actors to share a common vision, and setting up systems for communication, coordination and conflict resolution [1,3,9].

While some authors [5,11,24] have a visual representation of their framework, showing the relationship between elements, some frameworks are descriptive and do not show the interaction and link between elements. We also noted that some elements described in the frameworks using different terms, most times refer to the same thing. For example, the administration element in the process framework [26] is referred to as backbone support in the collective impact framework [25], and coordination committee in Salunke and Lal [1]. Also noted as a gap in the existing frameworks is that most authors, other than Rasanathan, Bennett [3], draw lessons and conclusion about mulitsectoral action based on a generic instead of a specific case. Furthermore, only one framework [41] provides evidence of being developed and applied to a case study (e.g. Togo and South Africa). The proposed framework responds to some of the limitations (and other gaps highlighted above), as it draws lessons about MSA on a case of HIV in South Africa.

## Proposed framework on multisector multilevel collaboration

**Figure 2.**
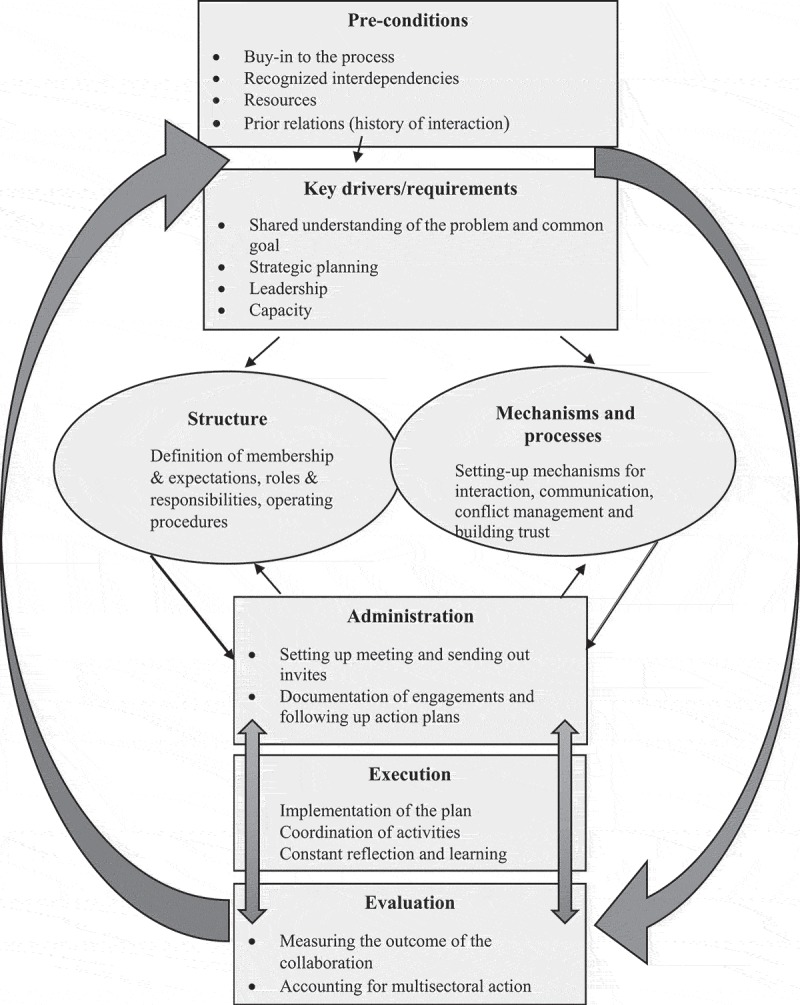
Framework for multisector and multilevel collaboration.

The framework builds on the existing literature, and distinctively draws from the challenges of implementation of the multisector approach that are described in Tables 1 and 2. We describe seven key components critical in a process of multisector collaboration including preconditions, key drivers, mechanisms, structures, administration, execution and evaluation. Figure 2 depicts the relationship between elements or components.

### Pre-conditions

Certain preconditions are critical in determining success or failure in multisector collaboratives.

***Buy-in to the process of collaboration*** is a crucial factor in driving implementation (or not) and ultimately determines success. While the donor community (World Bank and International Monitory Fund) played a critical role advocating for adoption of the multisector approach on HIV in most African countries, implementation was poor in some countries where there was lack of country ownership of the process. Buy-in to implement multisector action and ownership of the process is essential amongst stakeholders and sectors involved in a collaborative.

***Recognised interdependencies*** amongst sectors should be one of the preconditions for establishment of the collaborative. Participants must recognise their interdependence in moving towards a common goal or vision (Booher, 2004: 44). Bryson, Crosby [5] used the concept of sector failure to explain rationale for formation of collaboration, describing a state where single sector effort to solve a public problem have been implemented and failed and there is recognised need to work with others. Sector failure present as a window of opportunity for establishment of collaboratives [5]. In taking forward Bryson’s analysis, we also suggest the need for assessment of sector strengths and weaknesses to be a determinant and precursor for joint-action. As shown in our case study, a perception and attitude of self-sufficiency by sectors is an obstruction to effective collaboration.

***Resources*** are essential to enable multisector action. Planning and budgeting to ensure that there is required human, financial resources to enable the collaborative to undertake its task is of critical importance. Effective implementation of the multisector approach requires sufficient resources [1]. Costing of these activities should be included in the operational plan of the collaborative. We learned from the case study how some AIDS Councils struggled to function without resources, while those that had resources were much more effective. The case study also highlighted the importance of having a leader with capacity to mobilise resources for effective functioning of the collaborative. Having an influential champion with skills to negotiate and secure financial resources for a collaborative is an advantage [25]. Furthermore, budgeting for activities of the collaborative should be a multisectoral activity, undertaken by all sectors (stakeholders) involved. Each sector should indicate, in their action plan, resources that will be allocated to fund activities of the collaborative.

***Prior relations*** between sectors is important for multisector action [11]. It can have both a negative and a positive effect as a precondition for collaboration. Collaborations that have had prior interaction are able to judge trustworthiness of other parties and make an informed decision regarding their participation [5]. Those who have had good prior experiences will start from a positive stance, with confidence on what a collaboration is likely to produce. We also learnt that prior history can stifle effective functioning of collaboratives, as it was evident in the case study where civil society struggled to adapt to its new role as a partner in the collaborative. From time to time, civil society reverted to their past role (during the apartheid era), where the state-civil society relations tended to take an adversarial form, with civil society being a watchdog to the state [42]. Prior relations were in this case not good for effective functioning of the collaborative. Acknowledging and addressing such tensions should be prioritised.

### Key drivers/prerequisites

A shared understanding of purpose, common vision, leadership and capacity are critical for multisector action.

***Shared understanding of problem and a common vision is*** amongst the key prerequisites for multisectoral action [8,9]. Successful collaboration requires clarity of objectives and aim before the commitment to work together. All the stakeholders involved should have a common vision and perspective of what the collaborative aims to achieve [1]. Having a common understanding requires that stakeholders engage in a process of negotiation, which should be open to differing perspectives at first, and allow everyone space to seek clarity, and to express their concerns. Individual sectors should be able to connect and be clear on their role (including how they will benefit) in the bigger schism of the collaborative, which according to Hardy, Lawrence [43] will give them a reason to participate. Communication of expectations is important at this early stage in the process to manage expectations. In the case study, we found that members had varied expectations and aims which affected their commitment at a later stage when they realised that their expectations were not met. Negotiation skills are needed in the process of defining a common vision [9]. Open communication and willingness to compromise is equally important.

***Strategic planning*** resulting in a plan for multisector action with defined and concrete steps on how to achieve the objectives of the collaborative is amongst the key pre-requisite for multisector action. The plan should specify the objectives and activities, timelines, outcome measures, costs and an indication of how the plan will be financed. A process map may be useful to visually describe the flow of activities linked to the timelines [44]. A plan will also guide monitoring of progress and evaluation of outcome [1].

***Leadership****i*s a critical enabler in any implementation process [45–47]. There is agreement amongst scholars about the need for facilitative leadership that will create an enabling environment for effective collaboration [11,20,24,48]. A leader will be someone who will drive the collaborative agenda, and such a role should be undertaken by someone with credibility, and power to convene and to influence other sector’s decision regarding which programmes are prioritised [5,22]. Allocation of this role to political leaders – as was the case in the case of AIDS Councils – yielded positive results in that they were able to secure financial resources, and advocate for integration of HIV in programmes and activities of government. Having a political leader endorsing the process attracted support from other stakeholders.

A leader of the collaborative should be carefully chosen, as he or she has an influential role of chairing and steering the discussion during meetings [25]. Thus, needing to be well vested with the mandate to ensure that activities are aligned to the purpose and aim of the structure. Having an individual instead of a sector driving the collaborative should also be cautioned as the structure ceases to exist when individuals resign. Leadership of the collaborative should be linked to both an individual (who will be a representative), and to an organisation for continuity. It is also critical that other sectors beyond government are involved in the leadership role to encourage ownership and commitment by all sectors.

***Capacity of members*** is critical for the collaborative to function optimally [8]. Ideas, and innovation needed in addressing the problem and achieving the aim of the collaborative lies with the people whom together can explore strategies that are best suited to address a complex problem. Process skills including listening, negotiation and reasoning are crucial skills required by members of the collaborative to make a meaning contribution, and to effectively represent their sectors. While some of these skills are learned through experience, deliberate efforts should be facilitated by the leader of the collaborative to ensure that members have the skills and capacity.

### Structure

The design of the structure is one of the two central components of this framework (together with mechanisms) that are critical to the process of multisector collaboration.

***Membership and expectations*** are most often not clarified in collaboratives. The multisectorality of the structure should be clearly defined to inform recruitment and invitation of stakeholders to join. Composition of membership should be informed by a stakeholder analysis to ensure that the collaborative is constituted by the right people. Furthermore, representatives of sectors should be people with decision-making powers in their organisations, who will be able to implement resolutions from the collaborative. It is also important that one or two people are appointed to represent their sector, instead of sectors sending different faces, which can compromise continuity. There is also a need to clarify what is expected by members, and their respective sectors to ensure alignment with the vision of the collaborative.

***Roles and responsibilities*** between sectors and individuals need to be clarified for effective multisector action [1,3]. The roles and responsibilities should be linked to the mandate of the collaborative and be measurable. Clarification of roles becomes easier when there is clarity about the comparative advantage each sector brings to the collaborative, and this is a process that requires deliberation. When roles and responsibilities are not clarified and communicated, there is a possibility of shifting of responsibility amongst members. Establishment of committees where individual members (and not only their sectors) will be allocated responsibilities is critical to ensure that everyone is clear on what is expected of them as a member of the collaborative. A written agreement on roles and responsibilities would greatly enhance the functioning of the collaborative.

***Standard operating procedures (SOPs)*** should be defined given that collaboratives bring together diverse members representing sectors with different backgrounds, and ideologies [3,5,26]. It is essential that the collaborative defines rules, and norms that will govern interactions between members. This process should be undertaken jointly with all stakeholders involved to facilitate compliance. SOPs are useful to help set boundaries, and define acceptable conduct in a collaborative.

### Mechanisms and processes

It matters how the practice of collaboration is carried out [48]. Central to the framework is establishment of mechanisms for collaboration, which are most often neglected in the literature [5]. Mechanisms are critical in driving the process of collaboration, beyond establishment of structure.

***Regular interaction*** including face to face meetings and other forms of engagement are critical for deliberations and for sectors to share knowledge and expertise. The meetings need to be planned, and be agreed upon by all stakeholders involved. Meeting regularly is also important to identify issues and concerns that might arise, and to address them. Planning meetings in advance is critical to secure commitment and participation from stakeholders. The content of the meetings should be pre-defined and linked to the vision of the collaborative. We learned from the case study that if there is no focus, structure and stimulating discussion during meetings, members are likely to lose interest, view them as a waste of time, and not attend the following meeting.

***Communication*** is critical for multisectoral action. Effective collaboration is not just about bringing sectors together, but occurs when sectors engage in meaningful participation [15,43]. Meaningful participation is dependent on how people interact, and require the use of communicative practices that will allow for management of diversity and tension that is immanent in collaboratives [15]. There is consensus in the literature about the importance of open communication, dialogue, and confrontational deliberation as critical communicative practices for meaningful participation [15,49,50].

Lack of confrontational deliberations that was observed in the case of AIDS Councils led to rising tension, which was not communicated nor resolved, consequently affecting meaningful contribution by some members of the collaborative. Collaborations are enacted in a series of conversations between people, and continued conversation is necessary if collaborative action is to ensue [43]. While communication is important, scholarly literature also cautions against the likelihood of power dynamics that are immanent in communicative processes [16,22,51]. A communication plan needs to be defined, and should include strategies to manage power dynamics in conversations.

***A conflict management plan*** to address tension inherent in multisector collaboratives is important, given that collaboration describes a process that brings together sectors from different background to constructively search for solutions that go beyond their own limited vision of what is possible [1,52]. Power disparities, bias (and dominance), and conflicting expectations are some of the possible sources of conflict [22,53,54]. A collaborative will need to predefine the process to resolve conflict, which should be known to all stakeholders involved. A safe space and platform to discuss areas of conflict is needed, and conflict should be embraced as an intrinsic part of the process of multisector collaboration. Timely remedial action should be taken [1].

***Trust*** helps drive and forms the basis of effective multisectoral collaboration [3,8,26]. Low level of trust is likely to result to low level of commitment [11]. Trust is either pre-existing when sectors form the collaborative, based on prior relations, or builds over time as members work together [5]. Small wins throughout the process will generate trust and commitment to continue to work towards achieving the bigger goal of the collaborative [43,48]. Applying the principles of good governance including transparency, fairness and equality is crucial to build trust in collaboratives. Sharing information and knowledge and demonstrating competency, good intentions, and execution of commitments are other strategies that can be used to build trust [5,26].

### Administration of the collaboration

Every collaborative has an element of administration required to enable effective functioning. Both financial and human resources are needed for administration of the process of collaboration.

***Setting up and inviting members to meetings*** is an administrative task that requires someone to do it. Activities that are involved here include making telephone calls, sending emails to remind members about the meeting, and making logistical arrangements for the meeting to happen. We also learned from the case of AIDS Councils that an invitation to attend meetings carried more weight if it came from an office of someone with authority and influence. As such, we recommend that all invites and other communications have the signature of the leader, who will work together with an administrative team to oversee the process.

***Keeping record of meetings and follow-up on action plans*** are also some of the tasks that will be undertaken by the administrative team working together with the leader, and the process should be institutionalised to avoid reliance on one individual, and having the structure collapse if he or she resigns from the collaborative.

### Execution

Carrying out or execution of the plan is a step on its own, and a critical and challenging process. This step requires following the process map to ensure that objectives are implemented according to the set-timelines, and within the planned budget.

***Coordination*** is a critical process element, required to harmonise efforts of sectors involved. Coordination is even more essential when it involves sectors at multiple levels to avoid duplication and inefficient use of resources. Drawing from the case study of AIDS Councils, we also learned that effective coordination requires legitimacy and authority. For a collaborative to coordinate effectively, the structure should be recognised by those inside and outside, and needs to have authority and legitimacy to hold others accountable [5]. A team should be appointed to oversee the coordination process [1], and should work closely with the leader. Having one person solely responsible for this task has been cumbersome for coordinators in our case study. Capacity building is also critical to ensure that the coordinating team has the skill needed to undertake the task.

***Constant reflection and learning*** is important because collaboration happen in context. The framework acknowledges the likelihood of deviation that could be influenced by both internal and external factors. Emerson and colleagues [24] alluded to the critical role of the contextual environment in determining success or failure in collaboratives. A plan for constant monitoring and reflection is critical to manage the process, and to address environmental factors that might have a negative impact on the outcome [1].

### Evaluation

Measuring the outcome in collaboratives has been done using various approaches. Innes and Booher [55] propose that outcome should be assessed using first, second and third order effects; while Willis et al. [18] suggest measuring immediate, intermediate and long-term effects. While these authors use different terms, they both describe the timing when collaboration can be evaluated, and what should be realistically expected at that point. The first order or immediate effects refers to direct results of the collaboration, second order or intermediate effects refer to evaluation conducted when the collaboration is well underway, and third order focusing on long-term effects [18,48]. Similarly, Parkhurst and Preskill [56] describes three approaches to evaluate collective impact which are: developmental evaluation at the beginning, formative evaluation during the process as the collaborative evolves, and summative evaluation to assess the outcome at the end of the process.

Others suggest that collaborations should be judged on whether they produce public value, and that the judgement should be from a perspective of various stakeholders and not just the collaborative itself [6,16,19,24]. Public value can only be measured in a long-term, which we argue, if it becomes the only focus, the collaborative will miss the opportunity to celebrate ‘small wins’ – during the process – which are critical for building trust [11]. We suggest that the outcome is assessed at various points, and the scope of/or standards for evaluation should be defined at the onset, during the formation of the collaborative. The framework also acknowledges the possibility of unintended outcomes that might result from the process, which we assert are also important learning’s to inform future agenda on collaboration. Hence the link between evaluation, preconditions and key drivers is shown in Figure 2.

***Accounting for multisectoral action*** in joint initiatives pushes the boundaries of conventional accountability practices [57]. Collaboration is an entity, some of which are accountable to either government, donors or whoever has mandated its establishment, while some are stand-alone entities. Systems need to be put in place for reporting on progress against the goal, including a reflection on challenges and how they will be addressed. Accountability is an essential governance element in collaboratives, critical for building trust and enhancing effectiveness [58]. The leader will need to oversee and ensure that the collaborative is accountable through reporting and through other forms of accountability.

## Discussion

Implementing a multisectoral approach in practice is challenging, and the process compounded by many fundamentals that are needed to make it work. Despite the challenging nature of implementation, the multisectoral approach remains a critical strategy for addressing complex societal problems, as it allows for innovation and integrated efforts of multiple sectors [1,59]. What is needed is to find ways to make it work in practice. Effective implementation of a multisectoral approach is needed to enable achievement of the ambitious target of ending AIDS by 2030, and to achieve the ‘90–90-90’ treatment targets set by UNAIDS for 2020 [60,61]. While progress has been made in addressing various aspects of the epidemic, meeting the 90:90:90 targets requires innovative ways of engaging across sectors.

The framework builds from a suite of existing frameworks, models and approaches multisectoral action, and on the experience of implementation of the multisectoral approach on the response to HIV in South Africa. In this paper, multisector collaboration is presented as an iterative process that allow for improvement and learning. Highlighted in the proposed framework is that preconditions and drivers have an influence on the outcome of collaboration, and that the outcome of the collaborative process has an influence on the preconditions and drivers, and provides key lessons for the next process. Structure and mechanisms are the two central and interrelated elements of the proposed framework. The silo description of structure, observed in the literature, has been useful in stimulating ideas. However, we argue that it is limited without a connection to mechanisms. No matter how carefully designed the structure is, it cannot function effectively without the right mechanism; while mechanism alone will not be effective without a well-designed structure. The framework also alludes to the critical interconnection between administration, execution and evaluation components in the process of collaboration. Thus, addition of the two thick arrows running through the three components to depict connection between administration, execution and evaluation.

The framework is presented through a visual representation which shows how elements are connected, and how learning happens through-out the multisector collaboration process. By an indication of arrows, the framework describes the order in the process of multisector collaboration, including the feedback loops leading to the next process. The proposed framework is informed by empirical evidence from a case of implementation of a multisectoral response to HIV in South Africa.

Instead of focusing on one level, the framework draws from and reflects on the process of multisector collaboration that involves multiple sectors at multiple levels at national, provincial and local level. Collaboration becomes even more complex when there are multiple levels involved, and requires effective coordination. Sufficient human and financial resources are essential for effective coordination of the process. A team approach, skills and capacity are crucial for effective coordination of the multisector and multilevel collaboration. It should be coupled with guidance and support from a credible and influential leader who will drive the process, attract stakeholders to participate, and attract funding for effective implementation of activities of the collaborative. Multisector and multilevel collaboration is a governance process that can only effectively happen when key governance principles of responsiveness, participation, transparency and accountability are adopted and inform day-to-day engagements and deliberations.

The proposed framework should not be approached as static and rigid. It can be adapted taking into consideration the context and setting for which it is adapted for. Societal issues evolve and change over-time. They require constant reflection and reevaluation of strategies used to address them. The framework provides a road-map to help think about navigating the complex process of multisector and multilevel collaboration. It can be used by practitioners and policymakers to inform the design, implementation, monitoring, evaluation and for accounting on multisector collaborations. The framework is also a contribution to literature on MSA process, and can be used to inform theory development.

## Conclusion

The framework does not suggest that multisector collaboration on HIV is easy or a panacea as noted by others [6,16,62]. We, however, acknowledge and advocate for the need to find innovative ways to make collaborations to work, given that no one sector can adequately address the complex drivers of the epidemic. We also acknowledge that even with carefully designed collaborative processes, effective implementation will always be a challenge requiring effort and commitment to make it work [16]. As such, collaboration should be approached as an iterative process that requires flexibility, learning and modification, and should be informed by context. Further research is needed to test the applicability and effectiveness of the framework in strengthening multisector collaborations both in South Africa and in other countries with similar challenges that require a multisectoral approach.

## Paper context

Multisectoral action is often recommended as a strategy to address complex challenges in society, yet there is limited reflection on its implementation in practise. Existing frameworks on MSA mainly describe prerequisites, with limited explanation of MSA implementation process. A framework for multisector and multilevel collaboration is proposed to advance knowledge about process. Key elements are preconditions, drivers, structure, mechanisms and processes, administration, execution and evaluation. The framework can be used to inform theory, policy and practice.
